# Screening for differentially expressed circRNAs in ischemic stroke by RNA sequencing

**DOI:** 10.1186/s12883-021-02397-0

**Published:** 2021-09-25

**Authors:** Duncan Wei, Jian Chen, Xiaopu Chen, Shaoyan Wu, Zhaolin Chen, Yinting Huang, Zibin Shen, Wenzhen He

**Affiliations:** 1 Department of Pharmacy, The First Affiliated Hospital of Shantou University Medical College, No. 57, Changping Road, Guangdong 515041 Shantou, China; 2 Department of Neurosurgery, The First Affiliated Hospital of Shantou University Medical College, Shantou, China; 3 Department of Neurology, The First Affiliated Hospital of Shantou University Medical College, Shantou, China; 4 Shantou University Medical College, Shantou, China

**Keywords:** Ischemic stroke, CircRNA, High-throughput sequencing, Host gene

## Abstract

**Background:**

Ischemic stroke is a disease with high rate of death and disability worldwide. CircRNAs, as a novel type of non-coding RNAs, lacking 5’ caps and 3’ poly-A tails, has been associated with ischemic stroke. This study aimed to investigate key circRNAs related to ischemic stroke.

**Methods:**

RNA sequencing was performed obtain the circRNA expression profiles from peripheral whole blood of three ischemic stroke patients and three healthy individuals. Through bioinformatic analysis, differentially expressed circRNAs (DEcircRNAs) were identified, and GO and pathway analyses for the host genes of DEcircRNAs were conducted. The expression levels of selected circRNAs were analyzed with qRT-PCR. To further explore the functions of key circRNAs, a DEcircRNA-miRNA interaction network was constructed.

**Results:**

A total of 736 DEcircRNAs were detected in ischemic stroke. Functional annotation of host genes of DEcircRNAs revealed several significantly enriched pathways, including Fc epsilon RI signaling pathway, B cell receptor signaling pathway, and T cell receptor signaling pathway. The qRT-PCR results were largely in keeping with our RNA-seq data. The ROC curve analyses indicated that hsa_circ_0000745, hsa_circ_0001459, hsa_circ_0003694 and hsa_circ_0007706 with relatively high diagnostic value. A circRNA-miRNA network, including 1544 circRNA-miRNA pairs, 456 circRNAs and 4 miRNAs, was obtained.

**Conclusions:**

The results of our study may help to elucidate the specific mechanism underlying ischemic stroke.

**Supplementary Information:**

The online version contains supplementary material available at 10.1186/s12883-021-02397-0.

## Background

Stroke is a leading cause of long-term disability and life-threatening disease [1]. Ischemic stroke, which accounts for approximately 80 % of total strokes, results in reduced cerebral blood flow due to cerebral artery occlusion, leading to rapid loss of brain function [2]. However, at present, there is no effective treatment available for cerebral ischemic diseases; this deficiency is attributable to many factors, particularly the rapid development of brain injury following ischemia, molecular changes following acute ischemic stroke, complex interplays among signaling pathways, and the narrow therapeutic window for specific targets [3]. Hence, it is urgently important to elucidate the pathogenesis and underlying mechanisms of cerebral ischemic injury and develop effective treatment strategies for this disease.

Lacking 5’ caps and 3’ poly-A tails, circular RNAs (circRNAs) are resistant to RNaseR treatment (which degrades essentially all linear forms of RNAs), which makes circRNAs more stable than linear RNAs [4]. In addition, circRNAs are evolutionarily conserved among diverse species [4]. These features enable circRNAs to perform a variety of physiological functions, including mediating alternative splicing of mRNAs, regulating the transcription of parental genes and binding to miRNAs as competing endogenous RNAs [5]. Three models of circRNA regulating the expression of parental gene: (1) The intron-only ciRNA binds to elongating RNA Pol II and promotes host transcription in a cis-acting manner; (2) EIciRNA binds to U1 snRNP through specific RNA-RNA interaction between U1 snRNA and EIciRNA, and then the EIciRNA-U1 snRNP complexes might interact with RNA Pol II transcription complex to promote host gene expression; (3) CircRNA shares some miRNA binding sites with 3’-UTR of the transcript from their parental gene, and then CircRNA acts as miRNA sponge and increases the translations of the transcript from its parental gene [6].

CircRNAs have been reported to confer functions in multiple pathogenic processes, including cancers and stroke [7]. Bazan et al. reported that a high ratio of serum circR-284 to miR-221 is observed in acute ischemic stroke patients, indicating the potential diagnostic value of circRNAs in cerebrovascular ischemia [8]. Bai et al. indicated that significantly decreased circDLGAP4 was present in both acute ischemic stroke patients and a mouse stroke model [9]. Three circRANs (circFUNDC1, circPDS5B, and circCDC14A) were reported to be positively correlated with infarct volume, suggesting the 3 circRNAs may be envisioned as potential biomarkers for acute ischemic stroke diagnosis [10]. Li et al. suggested that hsa_circ_0000607 may play a crucial role in the pathogenesis and progression of acute ischemic stroke by regulating the miR-337-3p/Bcl2 axis [11]. Significantly decreased CircSCMH1 in the plasma of patients with acute ischemic stroke is of great significance in predicting stroke outcomes [12].

In the current study, we employed high-throughput RNA sequencing (RNA-seq) to investigate the circRNA expression profiles of ischemic stroke patients. Bioinformatic analysis was applied to identify differentially expressed circRNAs (DEcircRNAs) and DEcircRNA-miRNA interaction networks. In addition, the expression levels of selected circRNAs were validated with quantitative real-time polymerase chain reaction (qRT-PCR). By doing this, the results of our study may help to elucidate the pathogenesis and underlying mechanisms of ischemic stroke.

## Methods

### Patients and samples

The cohort subjected to RNA-Seq consisted 3 ischemic stroke patients and 3 healthy individuals. The etiology of stroke was classified according to Trial of Org 10,172 in Acute Stroke Treatment (TOAST) criteria [13]. Three patients with large-artery atherosclerosis were admitted at 2, 4, and 7 days after the onset of stroke, respectively. The inclusion criteria were ischemic stroke patients with initial onset aged 18–75 years who were diagnosed according to Magnetic Resonance Imaging (MRI) or computed tomography (CT) scan of the brain and clinical diagnostic criteria. Patients with other neurological diseases, including transient ischemic attack, cardiogenic cerebral embolism, hemorrhagic infarction, traumatic cerebrovascular disease, and occult cerebral vascular malformation, were excluded from the study. Healthy individuals were recruited from people who underwent a routine medical examination at the hospital. Individuals with a history of stroke, surgery, heart surgery, head trauma, or neurological disease were excluded. Table 1 describes the characteristics of these individuals. The subjects were matched for age and gender. All samples were collected after obtaining written informed consent. This study was approved by the ethics committee of the First Affiliated Hospital of Shantou University Medical College and performed in accordance with the principles of the Declaration of Helsinki. Peripheral whole blood (2.5 ml) drawn from each patient and each control subject was used for RNA extraction.

**Table 1:** Patient characteristics

	Case (*n* = 3)	Control (*n* = 3)	*p*-value
Age (mean (SD))	61.33 (8.39)	63.67 (4.51)	0.693
Gender = Male (%)	2 (66.7)	2 (66.7)	1
Hypertension = Yes (%)	2 (66.7)	0 (0.0)	0.386
Hyperlipidemia = Yes (%)	2 (66.7)	0 (0.0)	0.386
Diabetes = Yes (%)	1 (33.3)	0 (0.0)	1
Triglycerides (mean (SD))	2.09 (0.73)	1.21 (0.25)	0.122
HDL (mean (SD))	1.09 (0.14)	1.35 (0.31)	0.259
LDL (mean (SD))	3.62 (0.86)	3.63 (0.60)	0.979

*LDL* low-density lipoprotein; *HDL* high-density lipoprotein

### RNA sequencing and identification of DEcircRNAs

Using TRIzol reagent, total RNA was extracted from samples. RNA integrity and concentration were evaluated with an Agilent 2100 Bioanalyzer. Total RNA samples used in subsequent experiments fulfilled the following requirements: RNA integrity number (RIN) > 7.0 and 28 S/18S ≥ 1. In brief, total RNA was subjected to ribosomal RNA (rRNA) removal using the Ribo-Zero. To remove linear RNAs, total RNA was digested with RNase R. A total amount of 3 µg RNA was used for library preparation. Libraries for sequencing were constructed according to the manufacturer’s protocol. The quality of the libraries was determined using an Agilent 2100 Bioanalyzer and ABI StepOnePlus Real-Time PCR System. Libraries measuring 100–200 bp were selected. RNA sequencing was performed based on HiSeq 10X-150PE and 10 GB RNA-seq data per sample was generated. Low-quality data, adapter sequences and sequences with N base rate of raw reads > 1 % were filtered using SOAPnukev1.5.2 (parameters: -l 15 -q 0.2 -n 0.01 –i) [14]. Then, the remaining clean reads with high quality were subsequently aligned to the human reference genome (hg19) using BWA [15]. CIRI (v2.0.5) [16] uses BWA and find_circ (v1.2) [17] uses Bowtie2 (http://bowtie-bio.sourceforge.net/index.shtml) with default parameters to detect circRNAs, respectively. In this study, circRNA expression was calculated according to the junction reads count at both ends of the circRNA, and the final junction reads count takes the average value of the two software results. The junction reads per billion mapped reads were applied to normalize all samples. DEGseq, an R package to identify differentially expressed genes or isoforms for RNA-seq data from different samples, takes uniquely mapped reads from RNA-seq data for the two samples with a gene annotation as input [18]. With DEGseq v 1.43.0, DEcircRNAs were identified. Compared with controls, the DEcircRNAs in ischemic stroke were defined with *p*-value < 0.05 & |log_2_ FoldChange| > 2. Hierarchical clustering analysis of DEcircRNAs was performed with R package “pheatmap”. Then, enrichment analysis for host genes of DEcircRNAs was performed by GeneCodis3 with R package ggplot2.

### ***In vitro*** validation

Following the manufacturer’s protocol, total RNA was isolated from blood samples of 15 ischemic stroke patients and 15 healthy controls with the TRIzol reagent. RNA integrity and concentration were evaluated by NanoVue Plus. By using FastQuant cDNA (Tiangen, Beijing, China), we generated cDNA from 1 µg extracted RNA. The qRT-PCR analyses were performed in an ABI 7300 Real-time PCR Detection System with SuperReal PreMix Plus (Invitrogen, USA). The qRT-PCR thermal cycling parameters were as follows: an initial denaturation step of 15 min at 95℃, followed by 40 cycles of 10 s at 95℃ and 30 s at 55℃, 32 s at 72℃, and 15 s at 95℃, 60 s at 60℃, 15 s at 95℃. Relative gene expression was calculated with the 2^−ΔΔCT^ method. Statistical significance was assessed by t-test. GAPDH was utilized as an internal control. The characteristics of these individuals were presented in Table S1. We designed specific divergent primers spanning the back-splice junction sites of circRNAs. The PCR primers were displayed in Table 2.

**Table 2:** The primers used in qRT-PCR experiments

circRNA	Pimers
GAPDH	Forward: 5’ TCGACAGTCAGCCGCATCTTCTTT 3’
	Reverse: 5’ ACCAAATCCGTTGACTCCGACCTT 3’
hsa_circ_0001459	Forward: 5’ ACAGCCCAATACTCATCACCAG 3’
	Reverse: 5’ TCTTCACTCTTGAGCACTGAATCT 3’
hsa_circ_0007706	Forward: 5’ TGAAGAAGAAGAAGTCACCAAAGGA 3’
	Reverse: 5’ GCAAACTGTGGGAAAGCCAT 3’
hsa_circ_0000745	Forward: 5’ ATGTTGAAAGTAGCCCGAGCAG 3’
	Reverse: 5’ TGGGAGTGTTGGAAGAAGTTGG 3’
hsa_circ_0003694	Forward: 5’ AGCAGGAATTGAGCCACAGAG 3’
	Reverse: 5’ AGAAGCTGTAAAGGCCTGTTGT 3’
hsa_circ_0037852	Forward: 5’ TTCTCAGACCACATCCGCTG 3’
	Reverse: 5’ AGTCGCTGTTATCAGCTATTCTCT 3’

### miRNA prediction of validated circRNAs

Pathways with *p*_adj < 0.05 were defined as significantly enriched pathways. To further investigate the functions of circRNAs, the target miRNAs of circRNAs were predicted based on the RNAhybrid database (https://bibiserv.cebitec.uni-bielefeld.de/rnahybrid) with -sc > 150 and –en < 7. The circRNA-miRNA interaction network was visualized with Cytoscape (http://www.cytoscape.org).

## Results

### Identification of DEcircRNAs

**Fig. 1:**
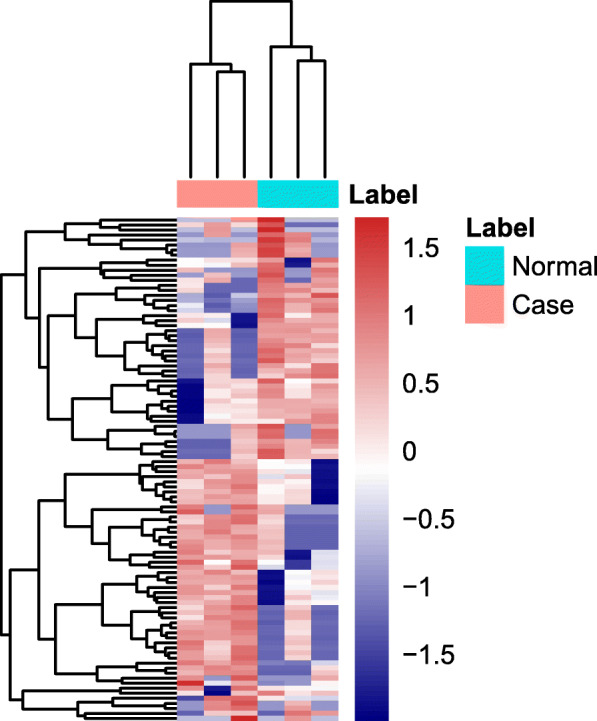
The heatmap of top 100 up-regulated and down-regulated differentially expressed circRNAs in ischemic stroke patients. Rows and columns represented DEcircRNAs and samples, respectively. The color scale represented the expression levels. Red and blue color indicated up- and down-regulation, respectively

A total of 22,434 circRNAs were detected in this study. Compared with normal controls, 736 DEcircRNAs (307 up-regulated and 429 down-regulated DEcircRNAs) were detected in ischemic stroke with *p*-value < 0.05 and |log_2_ FoldChange| > 2 (Fig. 1). These DEcircRNAs were widely distributed across almost all human chromosomes, including the sex chromosomes (Figure S1). Among these circRNAs, hsa_circ_0001459 and hsa_circ_0037852 were the most up-regulated and down-regulated, respectively (Table 3). The raw data have been deposited in the Gene Expression Omnibus database (GSE178764, https://www.ncbi.nlm.nih.gov/geo/query/acc.cgi?acc=GSE178764).

**Table 3:** Top 10 up- and down-regulated DEcircRNAs between patients with ischemic stroke and normal controls

GeneID	circBaseID	log_2_FC	*p*-value	Regulation	Host gene	N1	N2	N3	P1	P2	P3
chr4:178,274,461|178,274,882	**hsa_circ_0001459**	3.037662	0	up	NEIL3	0	0	253	1296	395	725
chr17:53,478,829|53,481,229	hsa_circ_0002015	2.576615	0	up	MMD	1059	108	92	1819	2522	4393
chr12:58,340,777|58,347,472	hsa_circ_0000412	2.298732	0	up	ATP23	648	0	391	1371	2061	2513
chr21:37,711,076|37,717,005	hsa_circ_0001189	2.129201	0	up	MORC3	130	496	460	1620	1052	2853
chr9:6,880,011|6,893,232	hsa_circ_0001839	2.1205	0	up	KDM4C	259	237	506	50	2390	2627
chr17:20,107,645|20,109,225	**hsa_circ_0000745**	2.025537	0	up	SPECC1	583	1444	529	8198	1096	2808
chr3:169,840,378|169,847,340	hsa_circ_0067900	3.239944	3.03E-277	up	PHC3	151	0	0	1096	110	453
chr18:196,636|199,316	**hsa_circ_0007706**	3.034998	9.43E-260	up	USP14	130	43	0	648	548	453
chr1:205,238,077|205,239,012	hsa_circ_0003344	2.098331	4.26E-231	up	TMCC2	43	172	230	1047	241	928
chr1:29,362,337|29,379,824	hsa_circ_0011173	3.928555	3.58E-223	up	EPB41	65	0	0	50	263	838
chr16:11,214,471|11,217,803	**hsa_circ_0037852**	-5.09647	0	down	CLEC16A	281	431	759	50	0	0
chr18:45,391,429|45,396,935	**hsa_circ_0003694**	-3.13386	0	down	SMAD2	562	625	1402	299	44	0
chr7:104,925,455|104,937,980	hsa_circ_0003656	-2.84711	0	down	SRPK2	540	970	965	199	88	113
chr15:50,330,964|50,339,661	hsa_circ_0035197	-2.41562	0	down	ATP8B4	1664	2478	2896	797	307	430
chr20:60,737,807|60,738,678	hsa_circ_0003231	-3.34158	1.31E-276	down	SS18L1	216	388	782	0	0	159
chr10:11,312,628|11,330,515	hsa_circ_0017680	-2.63557	8.20E-247	down	CELF2	1102	453	0	0	132	159
chr9:126,519,981|126,531,842	hsa_circ_0088474	-4.5753	1.71E-220	down	DENND1A	130	151	621	0	44	0
chr6:83,667,030|83,754,378	hsa_circ_0008236	-3.23452	9.18E-219	down	UBE3D	346	43	736	50	44	45
chr19:58,904,342|58,904,854	hsa_circ_0005598	-2.15791	9.64E-214	down	RPS5	605	474	621	0	307	136
chr17:20,910,208|20,914,622	hsa_circ_0042458	-3.45041	2.27E-212	down	USP22	238	129	667	0	110	0

*DEcircRNAs* differentially expressed circRNAs; *FC* fold change, *N1-3* normal controls; *P1-3* patients with ischemic stroke

### Functional annotation

**Fig. 2:**
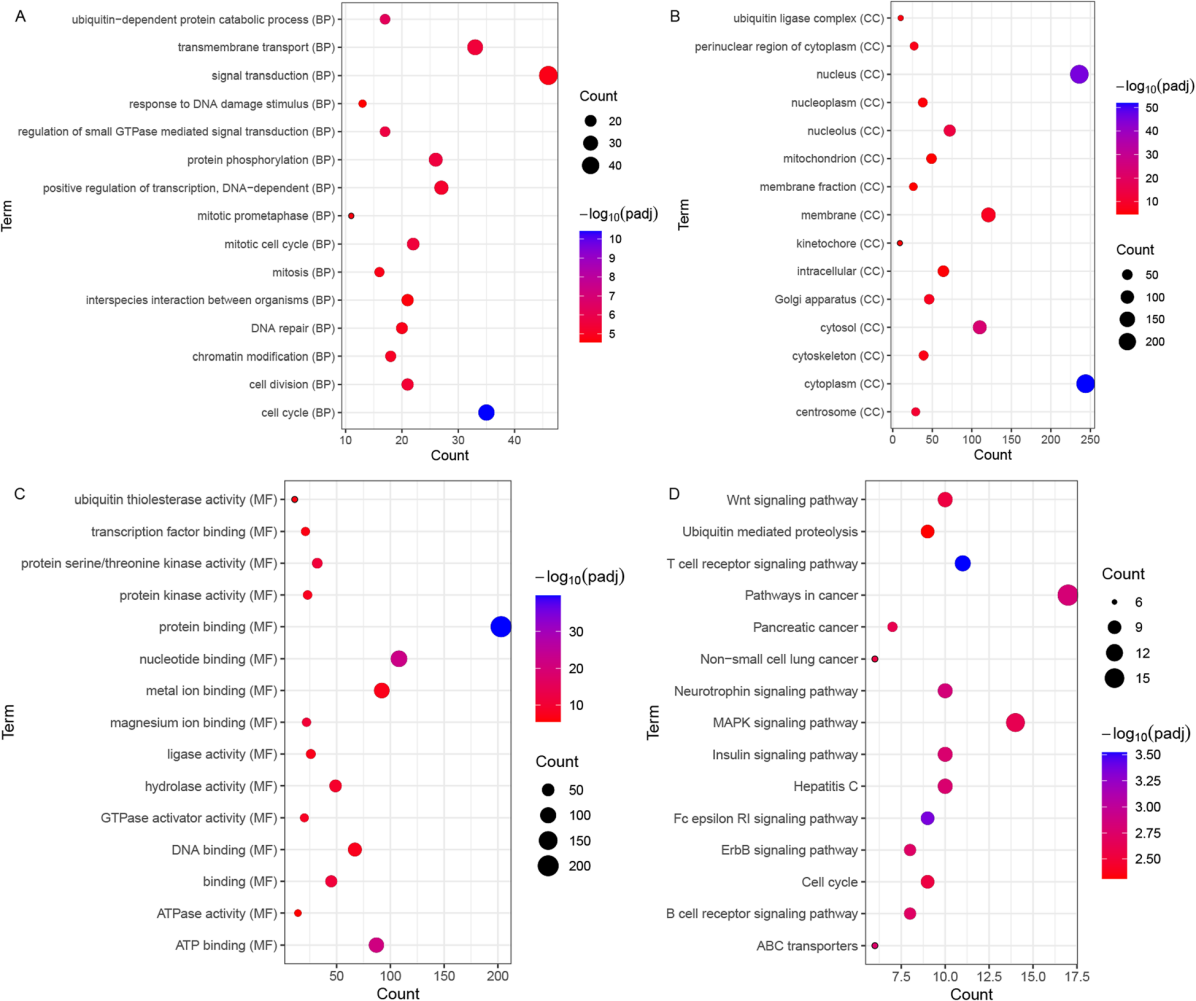
Significantly enriched GO terms and KEGG pathways of host genes of DEcircRNAs in ischemic stroke patients. (**A**) BP, biological process; (**B**) CC, cellular component; (**C**) MF, molecular function; (**D**) KEGG pathways. The x-axis shows counts of DEGs enriched in GO terms or KEGG pathways and the y-axis shows GO terms or KEGG pathways. The color scale represented -lg p_adj

In total, 607 host genes of DEcircRNAs were identified. GO analysis indicated several significantly enriched terms, such as, cell cycle (*p* = 2.65E-14), ubiquitin-dependent protein catabolic process (*p* = 7.03E-10), cytoplasm (*p* = 3.74E-55) and protein binding (*p* = 3.08E-43) (Fig. 2 A-C). KEGG pathway enrichment analysis indicated that several pathways were significantly enriched, including T cell receptor signaling pathway (*p* = 1.92E-06), Fc epsilon RI signaling pathway (*p* = 5.72E-06), B cell receptor signaling pathway (*p* = 3.72E-05) and Pathways in cancer (*p* = 3.93E-05) (Fig. 2D).

### ***In vitro*** validation

**Fig. 3:**
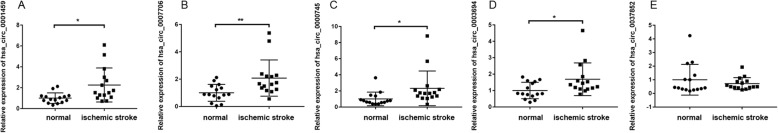
The qRT-PCR results of the DEcircRNAs in patients with ischemic stroke. (**A**) hsa_circ_0001459, (**B**) hsa_circ_0007706, (**C**) hsa_circ_0000745, (**D**) hsa_circ_0003694, (**E**) hsa_circ_0037852. * represented *p* < 0.05, ** represented *p* < 0.01

**Fig. 4:**
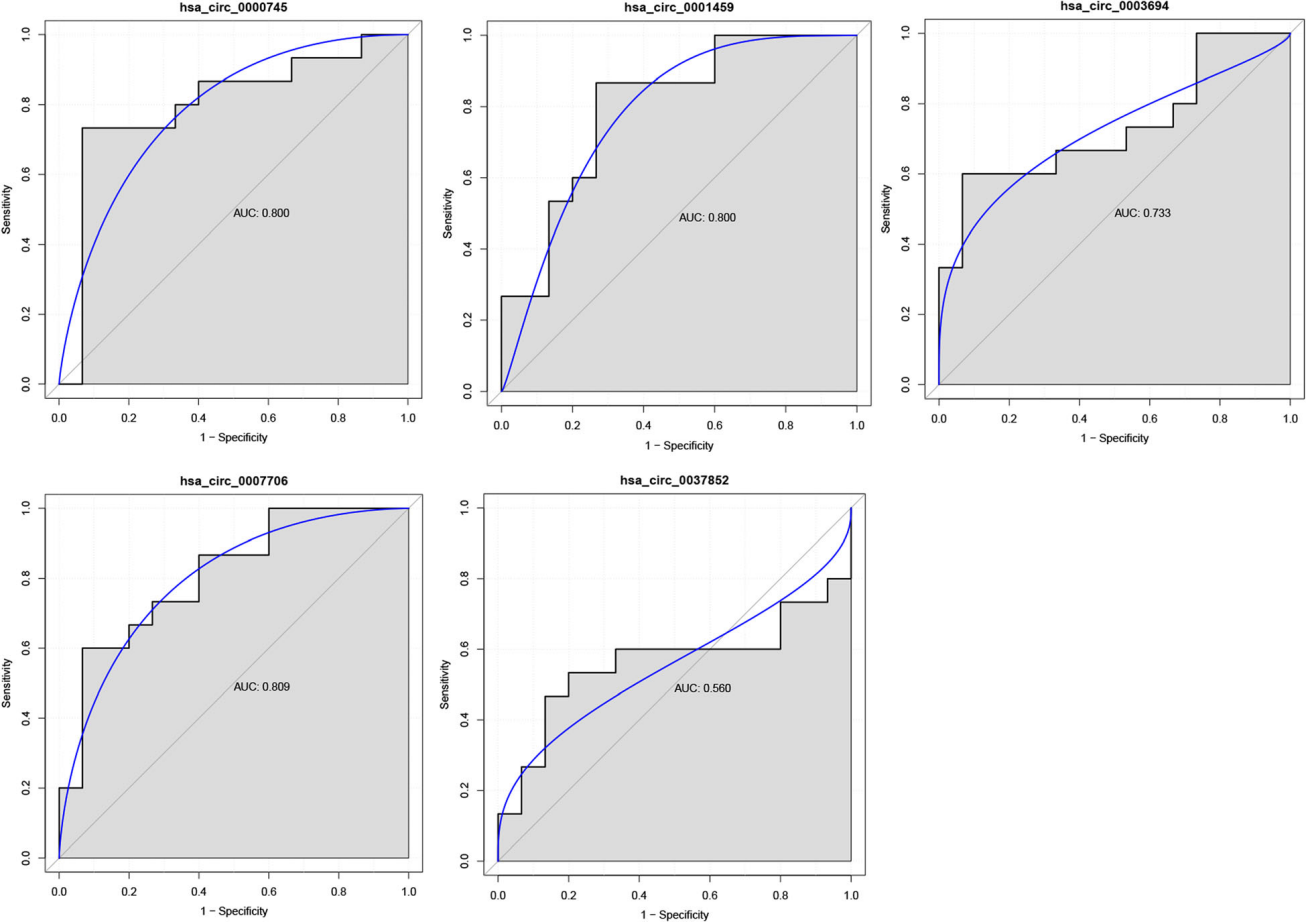
The ROC curves of DEcircRNAs in ischemic stroke. (**A**) hsa_circ_0000745, (**B**) hsa_circ_0001459, (**C**) hsa_circ_0003694, (**D**) hsa_circ_0007706, (**E**) hsa_circ_0037852

Five circRNAs, specifically three up-regulated (hsa_circ_0001459, hsa_circ_0007706 and hsa_circ_0000745) and two down-regulated DEcircRNAs (hsa_circ_0003694 and hsa_circ_0037852), were selected randomly from the top 10 dysregulated circRNAs (two were the most dysregulated, and three were randomly chosen) for qRT-PCR analysis. Three of these circRNAs at the level of significance and a trend in the same direction was observed for another. In other words, except for hsa_circ_0003694, the expression of the others in the qRT-PCR results generally exhibited the same pattern as that in our RNA-seq results (Fig. 3). Then, by using pROC package in R language, we performed the ROC analysis to assess the diagnostic value of DEcircRNAs. The area under the curve (AUC) was further calculated. And the ROC curve analyses indicated that the AUC of hsa_circ_0000745 (0.800), hsa_circ_0001459 (0.800), hsa_circ_0003694 (0.733) and hsa_circ_0007706 (0.809) was more than 0.7, and with relatively high diagnostic value (Fig. 4).

### DEcircRNA-miRNA interaction network

**Fig. 5:**
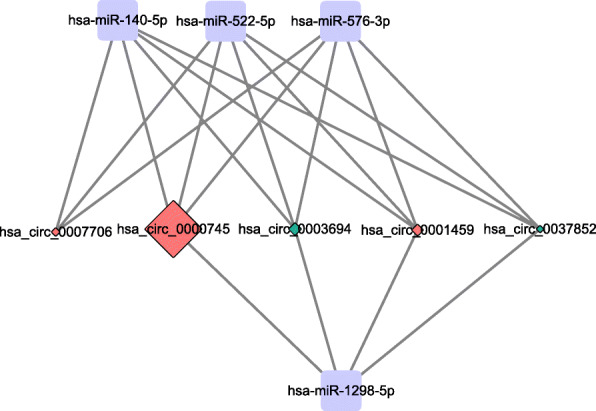
Subnetwork of hsa_circ_0001459, hsa_circ_0007706, hsa_circ_0000745, hsa_circ_0003694, and hsa_circ_0037852. The rhombic nodes and rectangle nodes indicate DEcircRNAs and miRNAs, respectively. Red and green color represent up-regulation and down-regulation, respectively. Nodes with black border were DEcircRNA derived from top 10 up-regulated and down-regulated DEcircRNA in ischemic stroke patients. The node size represents the expression level of circRNA. DEcircRNA, differentially expressed circRNA

A total of 1544 circRNA-miRNA pairs, involving 456 circRNAs and 4 miRNAs were obtained (Table S2). The subnetwork of hsa_circ_0001459, hsa_circ_0007706, hsa_circ_0000745, hsa_circ_0037852 and hsa_circ_0003694 were displayed in Fig. 5. In addition, NEIL3 was the host gene of hsa_circ_0001459, USP14 was the host gene of hsa_circ_0007706, SPECC1 was the host gene of hsa_circ_0000745, CLEC16A was the host gene of hsa_circ_0037852, and SMAD2 was the host gene of hsa_circ_0003694, respectively.

## Discussion

CircRNAs were once regarded as by-products of aberrant splicing, evolutionary junk or transcriptional noise with little functional potential [19]. Recently, these RNAs have attracted increasing attention, not only because of their conserved expression among species but also due to their functions. Ostolaza et al. suggested that hsa_circRNA_102488 may serve as an interesting candidate marker which showed a statistically significant change in expression between stroke etiology subtypes [20]. Lu et al. found that circBBS2 and circPHKA2 were differentially expressed in the blood of acute ischemic stroke patients and demonstrated that blood circRNAs may serve as potential biomarkers for acute ischemic stroke diagnosis [21]. In this study, a total of 736 DEcircRNAs were detected in ischemic stroke patients by high-throughput sequencing.

The hsa_circ_0001459, a circRNA derived from NEIL3, was identified to be up-regulated in both the RNA-seq results and qRT-PCR results in this analysis. Oxidative stress is known as one of the molecular mechanisms underlying cerebral ischemic stroke [22]. Chronic exposure to higher concentrations of reactive oxygen species (ROS) may cause damage to the DNA structures of brain cells, which results in an increased risk of stroke [23]. Nei like DNA glycosylase 3 (NEIL3) is a DNA glycosylase, that plays a vital role in repairing the DNA structure damaged by ROS [24]. Knockout of NEIL3 induces severe neuropathy, and NEIL3 knockout mice exhibited poor outcomes after ischemic stroke [25]. If cultured neural stem/progenitor cells lack NEIL3, the repair of oxidative DNA base lesions is significantly impaired [25]. Jalland et al. indicated that NEIL3 also promotes neurogenesis and reduces oxidative DNA damage to protect against prion disease [26]. The studies mentioned above suggested a close link between NEIL3 and ischemic stroke. Hence, we speculated that hsa_circ_0001459 may exert its effect by regulating its host gene NEIL3 in ischemic stroke.

Hsa_circ_0007706 was another significantly down-regulated circRNA in this analysis. Stroke is associated with over-production of misfolded and aggregating proteins [27]. Ubiquitin specific peptidase 14 (USP14), a deubiquitinated protein, negatively regulates proteasome activity and inhibits the degradation of ubiquitin-protein conjugates [28]. *In vitro*, IU1 inhibits USP14 activity to enhance proteasome activity, thereby reducing cell death induced by oxidative stress [29, 30]. IU1 is correlated with decreased protein aggregates and is thought to be a therapeutic target in the stroke model [27]. It has been suggested that miR-124 conferred a neuroprotective function by directly targeting USP14 after cerebral ischemia [31]. Taken together, these findings indicate that hsa_circ_0007706, derived from USP14, may be implicated in ischemic stroke.

It is worth noting that hsa_circ_0037852 is the most significantly down-regulated circRNA. C-type lectin domain containing 16 A (CLEC16A) has been shown to be highly expressed in immune cells, including B lymphocytes, dendritic cells and natural killer cells [32]. Fujimaki et al. indicated that CLEC16A may be a susceptibility locus for myocardial infarction in Japanese individuals without chronic kidney disease [33]. Yoshida et al. showed that the polymorphism of CLEC16A (rs9925481) was closely related to myocardial infarction in individuals without hypertension [34]. Similarly, hsa_circ_0037852, derived from CLEC16A, may also be involved in ischemic stroke.

Hsa_circ_0003694 was the second significantly down-regulated circRNA in ischemic stroke. In response to stroke, astrocytes convert to a reactive phenotype (known as reactive astrogliosis) [35, 36]. Reactive astrogliosis and glial scar formation are among the primary causes of the difficulty in achieving functional recovery after ischemic stroke, as their presence inhibits the regeneration of neurons [37, 38]. Zhang et al. suggested that RGMa suppresses neurological functional recovery and promotes reactive astrogliosis and glial scar formation, which was associated with SMAD family member 2 (SMAD2) [39]. Lu et al. indicated that GDF11 may promote the neurogenesis and angiogenesis after stroke, which was also related to SMAD2 [40]. SMAD2 is the host gene of hsa_circ_0003694, which was down-regulated in the RNA-seq results and up-regulated in the qRT-PCR validation. Hence, the precise role played by hsa_circ_0003694 in ischemic stroke has not been determined.

Hsa_circ_0000745 has been linked with various types of cancer. It was suggested that hsa_circ_0000745 was down-regulated in gastric cancer and considered as a diagnostic marker for gastric cancer [41]. Jiao et al. demonstrated that hsa_circ_0000745 could promote cervical cancer and was a candidate target for the treatment of cervical cancer in the clinic [42]. In this study, hsa_circ_0000745 exhibited increased expression in ischemic stroke, which was the first report to link hsa_circ_0000745 with ischemic stroke. However, further research on the correlation between hsa_circ_0000745 and ischemic stroke is required to confirm these findings.

The DEcircRNA-miRNA interaction network demonstrated that there was a shared miR-140-5p target in hsa_circ_0001459, hsa_circ_0007706, hsa_circ_0000745, hsa_circ_0037852 and hsa_circ_0003694. It has been suggested that angiogenesis is implicated in neurological functional recovery [43]. An *in vitro* study indicated that miR-140-5p inhibits angiogenesis after cerebral ischemia [44]. Sørensen et al. observed up-regulated miR-140-5p in the stroke group and indicated that it may be related to ischemic stroke [45]. It is widely recognized that circRNAs can regulate gene expression by serving as ceRNAs by sponging miRNAs. Therefore, these findings further highlighted the important roles of the DEcircRNAs mentioned above in the stroke.

In this study, GO and KEGG analyses predicted and analyzed the potential circRNA function and biological pathways. The stroke-related biological processes included the immune process (such as, T cell receptor signaling pathway, B cell receptor signaling pathway and Fc epsilon RI signaling pathway) and signal transduction pathways (such as, MAPK signaling pathway and Wnt signaling pathway). It is known that immunity is integral parts of the pathogenic processes provoked by ischemia and reperfusion [46]. Consistently, previous studies have been demonstrated that MAPK signaling pathway and Wnt signaling pathway were involved in ischemic stroke [47–49]. Our study is consistent with what is known about stroke-related pathology.

## Conclusions

In conclusion, to the best of our knowledge, these five DEcircRNAs were reported for the first time that may be associated with ischemic stroke by high-throughput sequencing in this study. In addition, these five DEcircRNAs are all intron-type, suggesting they regulate their host genes through the first mechanism described above. Inevitably, the current study has some limitations. First, the sample size for RNA-seq and qRT-PCR validation was small. Second, there are differences in the distribution of hypertension and hyperlipemia between these ischemic stroke groups and healthy control groups. Third, no significant differences in the expression levels of hsa_circ_0037852 in qRT-PCR were observed between ischemic stroke groups and healthy control groups and the opposite results between RNA-seq and qRT-PCR of circ-0003694 were observed. This discrepancy probably arose because of the relatively small sample size, technical bias, the differential distribution of hypertension, hyperlipemia between these two groups, and the heterogeneity among samples. More samples that strictly meet the requirements need to be involved in our research for further verification and functional experiments of the significance of circRNAs in ischemic stroke. In addition, the ratio of the circRNA to linear expression, the effect of polymorphism on circRNA, and the specific mechanism of circRNA regulation of its host genes would be included in our future work plan.

## Supplementary Information

**Additional file 1.**

**Additional file 2.**

## Data Availability

The datasets generated and/or analysed during the current study are available in the corresponding author on reasonable request. The datasets generated and/or analysed during the current study are available in the Gene Expression Omnibus database (GSE178764, https://www.ncbi.nlm.nih.gov/geo/query/acc.cgi?acc=GSE178764).

## Abbreviations

AUC: Area under the curve
circRNA: Circular RNA
CLEC16A: C-type lectin domain containing 16 A
CT: Computed tomography
DEcircRNA: Differentially expressed circRNA
MRI: Magnetic Resonance Imaging
NEIL3: Nei like DNA glycosylase 3
qRT-PCR: Quantitative real-time polymerase chain reaction
RIN: RNA integrity number
ROS: Reactive oxygen species
SMAD2: SMAD family member 2
TOAST: Trial of Org 10,172 in Acute Stroke Treatment
USP14: Ubiquitin specific peptidase 14
