# Supplementary material for: Screening for differentially expressed circRNAs in ischemic stroke by RNA sequencing
Source: BMC Neurol. 2021 Sep 25;21:370. doi: 10.1186/s12883-021-02397-0 (PMC8464123; doi:10.1186/s12883-021-02397-0)
Supplement: Supplementary file 1 — Additional file 1. [file 12883_2021_2397_MOESM1_ESM.docx]

**Screening for differentially expressed circRNAs in ischemic stroke by RNA sequencing**

**Duncan Wei^1^, Jian Chen^2^, Xiaopu Chen^3^, Shaoyan Wu^3^, Zhaolin Chen^4^, Yinting Huang^3^, Zibin Shen^3^, Wenzhen He^3, *^**

^1^Department of Pharmacy, The First Affiliated Hospital of Shantou University Medical College

^2^Department of Neurosurgery, The First Affiliated Hospital of Shantou University Medical College

^3^Department of Neurology, The First Affiliated Hospital of Shantou University Medical College

^4^Shantou University Medical College

^*^Correspondence: Wenzhen He, The First Affiliated Hospital of Shantou University Medical College, No. 57, Changping Road, Shantou, Guangdong 515041, China.

Tel: +86-13005232005

E-mail: wenzhen_he@sina.com

Table S1 Patient characteristics

|  | **Case (n=15)** | **Control (n=15)** | ***p*-value** | **Overall (n=30)** |
| --- | --- | --- | --- | --- |
| **Age (years)** |  |  |  |  |
| Mean (SD) | 62.4 (5.55) | 62.13 (4.84) | 0.891 | 62.27 (5.09) |
| Median [Min, Max] | 62 [52, 70] | 62 [54, 70] |  | 62 [52, 70] |
| **Gender** |  |  |  |  |
| Female | 4 (26.7%) | 6 (40%) | 0.292 | 10 (33.33%) |
| Male | 11 (73.3%) | 9 (60%) |  | 20 (66.67%) |
| **Hypertension** |  |  |  |  |
| Yes | 15 (100%) | 10 (66.67%) | 0.006 | 25 (83.33%) |
| No | 0 (0) | 5 (33.33%) |  | 5 (16.67%) |
| **Hyperlipidemia** |  |  |  |  |
| Yes | 8 (53.33%) | 2 (13.33%) | 5.18e-06 | 10 (33.33%) |
| No | 7 (46.67%) | 13 (86.67%) |  | 20 (66.67%) |
| **Diabetes mellitus type 2** |  |  |  |  |
| Yes | 7 (46.67%) | 5 (33.33%) | 0.273 | 12 (40%) |
| No | 8 (53.33%) | 10 (66.67%) |  | 18 (60%) |
| **Triglycerides (mmol/L)** |  |  |  |  |
| Mean (SD) | 2.05 (1.08) | 1.18 (0.58) | 0.015 | 1.64 (0.98) |
| Median [Min, Max] | 1.54 [0.69, 4.4] | 1.03 [0.42, 2.73] |  | 1.43 [0.42, 4.4] |
| **HDL (mmol/L)** |  |  |  |  |
| Mean (SD) | 1.02 (0.26) | 1.22 (0.23) | 0.036 | 1.12 (0.26) |
| Median [Min, Max] | 1 [0.66, 1.59] | 1.25 [0.72, 1.7] |  | 1.15 [0.66, 1.7] |
| **LDL (mmol/L)** |  |  |  |  |
| Mean (SD) | 3.39 (0.82) | 3.10 (0.83) | 0.359 | 3.24 (0.83) |
| Median [Min, Max] | 3.27 [2.13, 5.24] | 3.37 [1.1, 4.26] |  | 3.37 [1.1, 5.24] |

LDL, low-density lipoprotein; HDL, high-density lipoprotein.

Figure S1


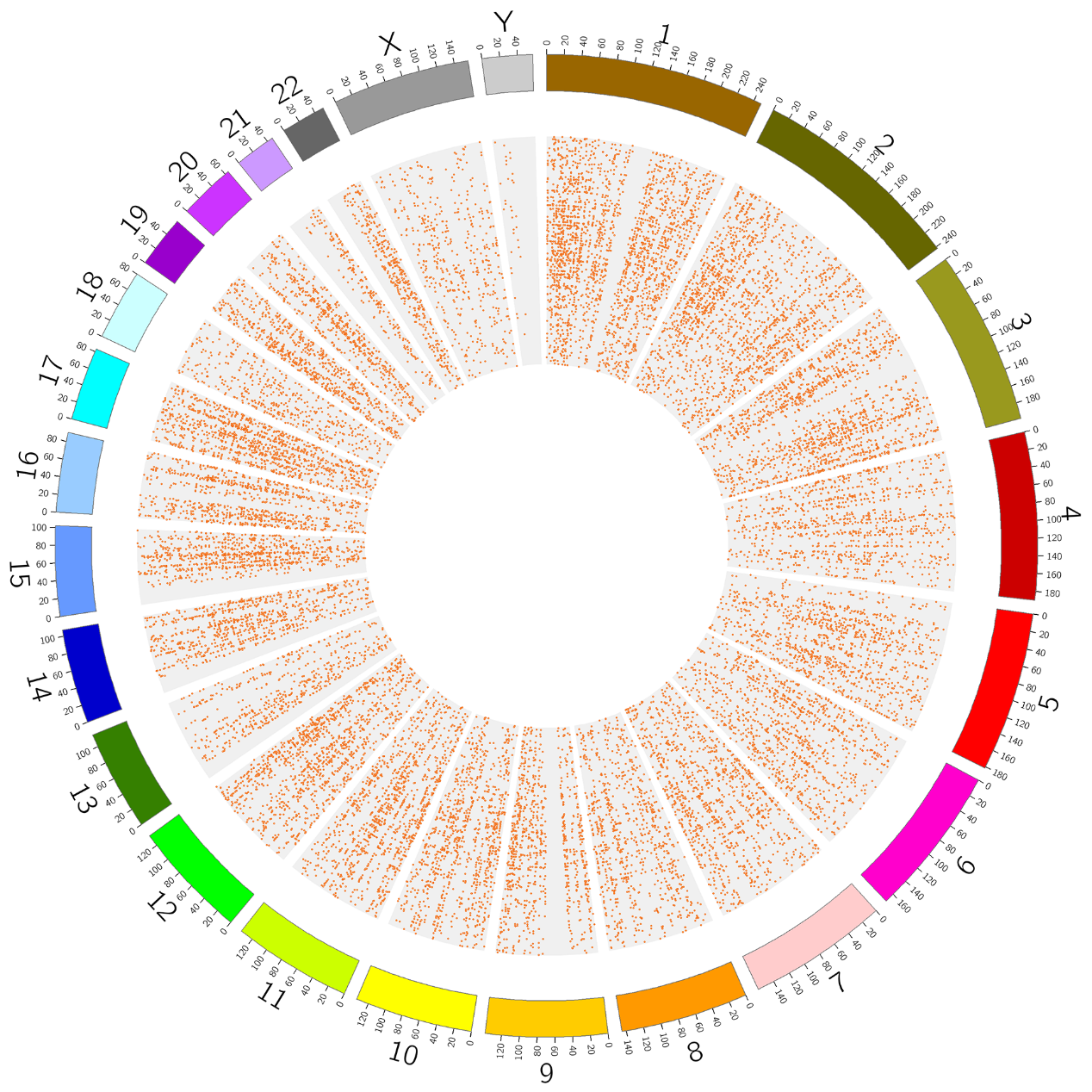


**Figure S1** The chromosome distribution of identified circRNAs.
